# Iron Supplementation in Suckling Piglets: An Ostensibly Easy Therapy of Neonatal Iron Deficiency Anemia

**DOI:** 10.3390/ph11040128

**Published:** 2018-11-22

**Authors:** Mateusz Szudzik, Rafał R. Starzyński, Aneta Jończy, Rafał Mazgaj, Małgorzata Lenartowicz, Paweł Lipiński

**Affiliations:** 1 Department of Molecular Biology, Institute of Genetics and Animal Breeding, Polish Academy of Sciences, Jastrzębiec, 05-552 Magdalenka, Poland; m.szudzik@ighz.pl (M.S.); r.starzynski@ighz.pl (R.R.S.); a.jonczy@ighz.pl (A.J.); r.mazgaj@ighz.pl (R.M.); 2 Department of Genetics and Evolution, Institute of Zoology, Jagiellonian University, Gronostajowa 9, 30-387 Kraków, Poland; malgorzata.lenartowicz@gmail.com; 3 Department of Genetics and Animal Breeding, Poznan University of Life Sciences, Wołyńska 33, 60-637 Poznań, Poland

**Keywords:** hepcidin, iron deficiency anemia, iron dextran, neonatal period, pig, supplementation

## Abstract

In pigs, iron deficiency anemia (IDA) is the most prevalent deficiency disorder during the early postnatal period, frequently developing into a serious illness. On the other hand, in humans, only low-birth-weight infants, including premature infants, are especially susceptible to developing IDA. In both human and pig neonates, the initial cause of IDA is low birth iron stores. In piglets this shortage of stored iron results mainly from genetic selection over the past few decades for large litter sizes and high birth weights. As a consequence, pregnant sows cannot provide a sufficient amount of iron to the increasing number of developing fetuses. Supplementation with iron is a common practice for the treatment of IDA in piglets. For decades, the preferred procedure for delivering iron supplements during early life stages has been through the intramuscular injection of a large amount of iron dextran. However, this relatively simple therapy, which in general, efficiently corrects IDA, may generate toxic effects, and by inducing hepcidin expression, may decrease bioavailability of supplemental iron. New iron supplements are considered herein with the aim to combine the improvement of hematological status, blunting of hepcidin expression, and minimizing the toxicity of the administered iron. We propose that iron-deficient piglets constitute a convenient animal model for performing pre-clinical studies with iron supplements.

## 1. Iron Deficiency Anemia (IDA) in Suckling Piglets

Iron deficiency is a very common condition, which is widespread in the human population [1]. In humans, individuals at increased risk of developing iron deficiency are young children, adolescents, and postpartum women. There is evidence in humans that iron deficiency at birth is relatively rare with the exception of low birth-weight-infants, including premature infants [2]. Pigs (*Sus scrofa domestica*) are the only mammalian species, in which neonatal iron deficiency anemia (IDA) commonly occurs, the most serious consequence of iron deficiency [3,4,5]. This disorder affects piglets of all breeds but its outcome is particularly severe in animals of high performance breeds. The occurrence of IDA in suckling piglets has been known for long time: The first case of anemia in piglets has been reported at the end of the 19th century [6], then in 1924 a causal link had been made between iron deficiency and the appearance of anemia [7]. Finally, 5 years later it was shown that anemia may be prevented by the administration to piglets of iron salts (FeSO_4_) [8]. The problem of neonatal IDA in piglets was first comprehensively drawn to the attention of the scientific community by Venn et al. in 1947 [3]. Thereafter, various aspects of IDA in piglets such as etiology, clinical aspects, diagnosis, prophylaxis and treatment have been the subject of several reviews [3,4,5,9,10,11].

IDA in suckling piglets is typically hypochromic, microcytic anemia characterized by a decrease in red blood cell (RBC) parameters such as mean corpuscular volume (MCV) and mean corpuscular hemoglobin concentration (MCHC). However, it is worth noting that physiological reference ranges for RBC indices of piglets in the early postnatal period (from birth to weaning, i.e., usually up to day 28 postpartum) commonly used to diagnose IDA are difficult to estimate. In swine production, iron supplementation of piglets is a routine and mandatory practice performed with the use of various iron supplements, administered by various routes, at differential doses of supplemental iron, and following various time schedules (reviewed in [9,10]). It is not surprising therefore that RBC parameters strongly vary in piglets depending on iron treatment protocols. On the other hand, without iron supplementation piglets rapidly develop IDA, and thus, by definition, RBC values measured in these animals (most frequently when pigs are used for research purposes) cannot be considered as normal ones. Nevertheless, there is a general consensus regarding hemoglobin concentration to set the cut-off level of anemia in suckling piglets at 8 g/dL [12,13], although some authors propose higher values reaching 11–13 g/dL [14]. It is clear from many studies [15,16,17,18,19,20] including our own [15,16,17] that such a level of Hgb is reachable only in piglets supplemented with very high doses of iron. Without iron supplementation IDA in piglets appears few days after birth. 1-day old piglets are usually not anemic showing Hgb concentration slightly above 8 g/dL [12,15]. It is important to underline that most iron supplementation therapies start on the 2nd or 3rd day after birth, therefore, this initial, sensu stricto normal Hgb value is a convenient starting point for testing the efficacy of new supplementation procedures in both veterinary practice and research. On day 3, Hgb concentration in non-supplemented piglets drops to the level of 6–7 g/dL and at weaning is drastically decreased even down to 4–5 g/dL, which indicates a state of extreme anemia [15,16,21]. Due to the efficient supplementation widely used in suckling piglets (see chapter below), anemia was practically eliminated from pig husbandry, however, in research, anemic piglets still constitute an indispensable reference group for exploring the molecular background of systemic iron metabolism in neonatal and early postnatal periods.

### Etiology of IDA in Suckling Piglets

Low iron stores. In most mammals iron accumulated during pregnancy in the fetal liver is the main source of this microelement for erythropoiesis and other iron-dependent processes in the neonatal period [22]. For example, infants born full term with an appropriate weight for gestation have iron stores that are adequate for their development during about half a year after birth [22]. In contrast, piglets are born with very limited iron reserves estimated at 50 mg, which are considered the lowest among mammalian newborns [3,4]. This reduced hepatic iron reservoir in pig neonates is reminiscent of that reported in low-birth-weight and preterm infants, congruent with the fact that the birth iron stores are generated primarily during the last period of gestation (last 10 weeks in pigs [23] and third trimester in humans [22]). In both humans and pigs, low iron stores are associated with increased risk of neonatal iron deficiency anemia. Iron content in the piglet at birth covers the requirement for only the first 3–4 days of postnatal life. Indeed, in non-supplemented piglets on day 4 after birth hepatic iron content is decreased by about 5-fold compared to day 1, and on day 7 is barely detectable [15]. In this context, the question why iron stores in normally born piglets are strikingly low arises. It is conceivable to propose that the main reason is the physiological inability of pregnant sow to meet iron demand for the greater number of fetuses. Unceasing improvement of the reproductive potential (including increasing litter size), is one of the objectives of modern pig husbandry. According to the Annual Report of the Polish Pig Breeders and Producers Association “POLSUS” the number of alive born piglets in two main Polish breeds (Polish Large White and Polish Landrace), increased by 0.5 and 0.7 between 2008 and 2017, respectively [24]. Such performance, which is an effect of intensive selection for reproductive traits, largely exceeds the physiological potential of sows to provide a sufficient amount of iron to their fetuses. Interestingly, wild boars (ancestors of domesticated pigs still present in the wild) having about half as many piglets in the litter as domestic pigs [25], seem to be not affected by neonatal IDA. Apparently, pigs domesticated from wild boars in Europe and Asia about 10,000 years ago [26] have not been able to evolve quickly enough to deal with high breeding pressure exerted only since the 19th century.

A second plausible explanation of deficient iron stores in pig fetuses and neonates is a low efficiency of iron transfer across the placenta. In this context, it is worth noting that iron supplementation of sows at various stages of pregnancy, using various iron supplements administered orally or parenterally has no significant impact on the improvement of the iron status of newborn piglets, and thus does not prevent suckling animals from becoming anemic (reviewed in [4]). The fact that despite iron abundance in pregnant sows, the iron status of their offspring still remains compromised, strongly suggests an insufficiency of the molecular machinery involved in iron transfer from mother to fetus. Obviously, this issue deserves further investigation. Our knowledge about placental iron trafficking and its regulation in pigs is poor. Surprisingly, even in mice and humans (in which iron metabolism is best studied among mammalian species) the placenta is considered the forgotten organ of iron transport, and regulatory mechanisms of iron transport from maternal to fetal circulation are only now emerging [27]. Moreover, when comparing placental iron transfer in various species, we should be aware of structural differences between human/rodent and pig placenta (hemochorial vs. epitheliorial type) because they may imply different regulations of iron trafficking across the placenta.

Low iron content in the sow’s milk. Low iron concentration in the sow’s colostrum and milk is commonly considered as risk factor in developing IDA in piglets [3,4,5]. Indeed, sow’s milk is a very poor source of iron containing about half as much of this microelement as human milk. Iron content of sow’s milk according to various reports ranges from 1.4–2.6 [28] to 0.2–4 mg/L [29]. Assuming that the daily milk intake per piglet is 0.5–1 L and that the absorption rate of milk iron is 60–90%, the piglet absorbs approximately 1 mg iron per day, an amount that is far below their daily requirement (about 7 mg) [3,30]. Importantly, attempts to increase the iron concentration in sow’s milk by giving them high iron diets or by the injection of large amounts of iron dextran during late gestation and lactation have not been successful [3,31].

**Figure 1:**
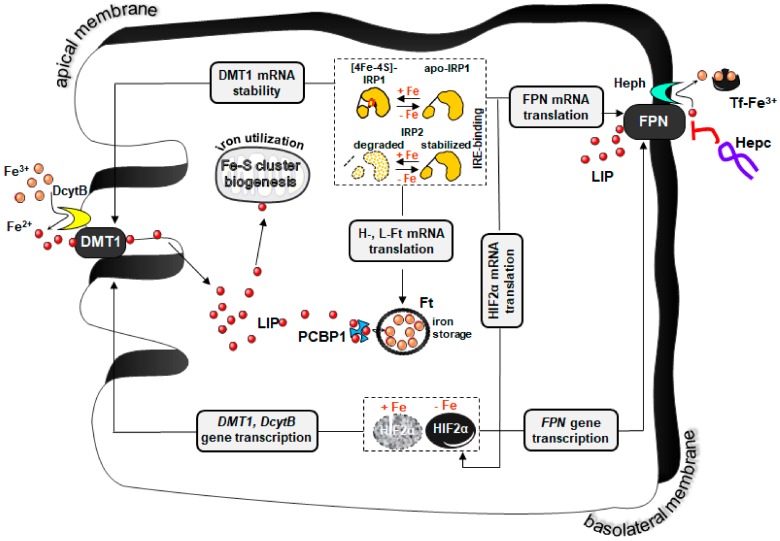
Intestinal uptake of non-heme iron and its regulation. Non-heme iron absorption occurs mainly in duodenal enterocytes. The first step in the transport of iron across the apical membrane of enterocytes is ferric (Fe^3+^) to ferrous (Fe^2+^) iron reduction, catalyzed by the membrane-associated ferrireductase duodenal cytochrome B (DcytB). Ferrous iron is subsequently transported into the enterocyte via the divalent metal transporter 1 (DMT1)-dependent pathway. After crossing the apical membrane, iron enters so called labile iron pool (LIP) in the cytosol and is subsequently used for cellular needs (e.g., for iron–sulfur cluster biogenesis in mitochondria), stored inside the cell in ferritin (Ft, which probably requires the chaperone PCBP1 (poly (rC) binding protein 1) to delivers iron to Ft), or exported into the circulation by the iron exporter ferroportin (FPN) present on the basolateral membrane. Iron export from enterocytes also requires hephaestin (Heph), a multi-copper oxidase, which oxidizes Fe^2+^ to Fe^3+^, prior to iron binding by transferrin (Tf) in the blood. The expression of genes involved in iron absorption is regulated intracellularly at the level of transcription by hypoxia inducible factor 2 alpha (HIF2α) and post-transcriptionally via iron regulatory proteins (IRP1 and IRP2). Under iron-deficient conditions, stabilization of HIF2α protein leads to the transcriptional up-regulation of *DcytB*, *Dmt1* and *Fpn* genes. In contrast, in iron replete enterocytes HIF2α undergoes accelerated proteosomal degradation resulting in the decrease of its transcriptional activity. At low intracellular iron concentrations, IRPs bind to specific iron regulatory elements (IREs) present in the 5′-UTR mRNAs encoding ferritin subunits (H- and L-Ft) or FPN mRNAs and block their translation. On the other hand, direct interactions between IRPs and several IRE motifs in the 3′-UTR DMT1 mRNA stabilize this transcript. The converse regulation of Ft subunits, FPN and DMT1 synthesis, being a consequence of the lack of binding of IRPs to IRE, occurs in enterocytes with high iron level. Importantly, the presence of IRE has also been identified in HIF2α mRNA. Binding of IRP1 (which under iron deficient conditions gains the ability to recognize IREs with high affinity) to the unique IRE in the 5′-UTR of HIF2α mRNA blocks its translation. Iron trafficking across the enterocyte is also controlled extracellularly by the systemic iron regulatory hormone hepcidin (Hepc). Hepcidin can bind to FPN, causing its internalization and degradation, hence decreasing iron export from enterocytes into the blood plasma.

Immaturity of molecular iron absorption mechanisms? Immaturity of the molecular machinery involved in intestinal iron absorption and its regulation in newborn piglets have been claimed as a factor contributing to the development of IDA [15]. Although our understanding of processes of dietary iron uptake in the neonatal period of mammalian development is poor, there are strong indications from studies on rodents that mechanisms of iron absorption clearly established in adult mammals are not fully functional during early life [32,33,34]. In adults, iron absorption occurs mainly in the proximal part of duodenum. At the cellular level, iron is absorbed through the polarized simple columnar epithelial cells located at the mid to upper villus. The passage of non-heme iron through the absorptive enterocyte from the gut lumen into the portal circulation involves two major steps: (1) Transfer across the enterocyte brush border (apical) membrane by the iron transporter–divalent metal-ion transporter 1 (DMT1) [35], a process preceded by the enzymatic reduction of the dietary ferric iron by duodenal cytochrome *b*, (DcytB, an apical membrane ascorbate-dependent ferrireductase) [36]; (2) export from the enterocyte across the basolateral membrane into intestinal capillaries via the sole iron exporter known to date—ferroportin [37]; the trans-membrane ferroxidase, hephaestin, colocalizes with ferroportin in the basolateral membrane and oxidizes the exported ferrous iron back to ferric iron, which is then complexed to transferrin [38]. Both, DcytB and hephaestin seem not to be indispensable for dietary iron absorption at least in mice, as animals with targeted disruption of the *Cybrd1* gene coding for DcytB show normal iron phenotypes [39], and in *sla* mice carrying a mutation in the *heph* gene, the transport of iron into the circulation is only diminished [38]. The efficiency of iron absorption is normally regulated in accord with iron status of the organism by hepcidin, a 25 amino acid peptide hormone released in to the blood plasma mainly by hepatocytes that negatively regulates the efflux of absorbed iron from enterocytes in the duodenum, the release of recycled iron from macrophages as well as the release of iron stored in hepatocytes [40]. This well-documented biological activity of hepcidin relies on its binding to ferroportin, which leads to its degradation [41]. Hepcidin is induced upon iron loading to decrease the iron level and in this way limits iron toxicity, whereas during iron deficiency there is increased erythropoietic activity and hypoxia, therefore, hepcidin expression is down-regulated to increase iron availability [40]. Recent studies demonstrated that the rate of dietary iron uptake is also locally controlled in absorptive enterocytes and that hepcidin-mediated regulation alone is insufficient to reduce iron absorption [42,43]. These studies showed the molecular mechanism of functioning of so called “mucosal block” [44], a concept of the regulation of iron absorption by duodenal ferritin, protein with an enormous capacity to store iron (up to 4500 Fe atoms/molecule [45]), which determines the level of basolateral iron efflux from the duodenal epithelium. Local control of duodenal iron absorption also involves transcriptional and post-transcriptional regulation of genes involved in iron absorption by hypoxia inducible factor 2 [46] and iron regulatory protein 1 [43], respectively. Recent advances in the understanding of this gut regulation have been detailed elsewhere [47]. In Figure 1, we provide an outline of duodenal non-heme iron absorption and its regulation.

It seems that in neonatal piglets fed almost an exclusive milk diet (mean daily intake of solid feed by piglets during two first weeks after birth is only slightly above 3 g [17]) and in infants with exclusive breast feeding, this complex molecular machinery described above is not mandatory for iron absorption. Lactoferrin, a major iron-binding glycoprotein abundantly present in milk, retaining bound iron down to a pH of ~3.5 [48], was postulated to be involved in intestinal iron absorption in suckling newborn animals and breast-fed infants. The identification of a specific receptor for lactoferrin (LfR) in the duodenum of newborn infants [49] and suckling piglets [50] is evidence that the Lf-LfR pathway is involved in iron absorption during early life. In this context, low duodenal expression of the two major iron transporters DMT1 and Fpn reported in pig neonates [15] may only impair absorption of iron contained in the solid diet and/or derived from dietary iron supplements and not Lf-bound iron, which uses an independent pathway to cross intestinal barrier.

High iron requirements in suckling piglets. Iron supply from natural sources such as hepatic iron stores and iron contained in the sow’s milk cannot balance the extremely high iron demand in suckling piglets. According to various authors their daily iron requirements are estimated at the level between 7 and 16 mg [20,30], which largely exceeds the amount of iron provided from natural sources. It is worth noting that upper reference values have been established for piglets with daily body weight gain of 250 g and more [20]. Indeed, high growth intensity of piglets in early postnatal life is a crucial factor determining this huge iron need. Piglets increase their body mass 2-, 4- and 10-fold within first week, 3 and 6 weeks after birth, respectively being the most rapidly growing animals among livestock species [5]. This impressive growth rate is accompanied by the expansion of blood volume, high erythropoietic activity resulting in an increased number of red blood cells that require an enormous amount of iron to maintain adequate hemoglobin level. Of note, a major part (~70%) of body iron is present associated with hemoglobin of developing erythroblasts in bone marrow and mature circulating erythrocytes.

## 2. Iron Supplementation of Piglets

### 2.1. Intramuscular Supplementation with Iron Dextran (FeDex)—A Gold Standard?

To counteract the development of anemia in young pigs, an exogenous iron source must be administered. Parenteral iron supplementation of piglets by intramuscular injection of 200 mg Fe in the form of FeDex (high molecular weight iron complex composed of a polynuclear iron hydroxide with dextran (polyisomaltose)) within 2–3 days after birth is routinely practiced in pig breeding and commonly considered by breeders and veterinary surgeons a golden standard for the prevention/treatment of IDA in suckling piglets [9]. Multiple variants of this method (in terms of number of injections, their time schedules and the amount of injected FeDex) have been proved to be generally beneficial in correcting iron deficiency in newborn piglets [15,18,20,51]. However, it seems that high parenteral intake of supplemental iron given in a single dose or even twice is not efficiently metabolized and may perturb the tight control of systemic iron homeostasis. First, excess of iron introduced to the organism may strongly exceed its capacity to store and detoxify this biologically active metal with ferritin. It is worth noting that after intramuscular injection of FeDex, this complex enters macrophages of the reticuloendothelial system (RES) via lymphatic circulation [52]. In macrophages, iron released from FeDex is either stored in ferritin [53] or redirected from these cells into the circulation by ferroportin where it is bound to transferrin and transported mainly to the erythropoietic compartment. In piglets supplemented with large amounts of FeDex, iron has been detected in Browicz-Kupffer cells (hepatic macrophages) by Prussian blue staining in the form of massive iron deposits [15]. Moreover, ferritin in the liver of these piglets has been found to be fully saturated with iron pointing to heavy pathological iron overload [15]. When in excess, iron is toxic because it generates through the Fenton reaction, the hydroxyl radical that reacts nonspecifically with biological molecules. Accordingly, piglets excessively loaded with FeDex showed increased hepatic levels of 8-oxo-7,8-dihydro-2′-deoxyguanosine (8-oxodG), an oxidatively modified nucleoside in DNA, a biomarker of iron-induced oxidative stress [15,54]. These piglets showed also elevated levels of 8-isoprostane, a biomarker providing a reliable measure of oxidative stress in whole organism [55]. It is not surprising therefore that an acute toxicity of the overdose of FeDex has been reported in antioxidant-deficient piglets [56]. Sporadically, toxicity of FeDex given to piglets may be also assigned to dextran (sugar part of FeDex)-induced anaphylactic reactions [57].

Second, applying FeDex therapy to piglets also implies the necessity for caution due to the possibility of excessive induction of hepcidin expression by supplemental iron, and in consequence, inhibition of iron absorption from the diet as well as iron release from macrophages (utilization of iron freed from FeDex). In humans, the incidence of such risk has been clearly demonstrated in iron-supplemented women showing significantly decreased fractional iron absorption, which was associated with acute increase in hepcidin [58]. In our studies, we have performed for the first time a comprehensive analysis of hepcidin expression (we measured the abundance of hepcidin mRNA in the liver as well as hepcidin concentration in blood plasma and urine) in suckling piglets supplemented with FeDex and in anemic, non-supplemented animals [16,17,59]. Administration of large amounts of iron to piglets resulted in a significant increase in hepcidin expression, which appeared immediately (only one day) after the injection of FeDex and was sustained at high levels up to weaning (day 28 after birth). In contrast, in anemic piglets hepcidin was hardly detectable in the blood plasma [16,59]. Our observation suggests that in piglets abundantly supplemented with iron, absorption of this microelement from the diet may be reduced. This could be a potentially unfavorable phenomenon especially in the second part of the early postnatal period (from day 14 following birth up to weaning), when voluntary intake of solid feed (containing usually high amounts of inorganic iron) by piglets is increased and the diet becomes a valuable source of exogenous iron. With the aim to correct iron deficiency in piglets without increasing hepcidin expression, we have proposed a modified procedure of FeDex administration, which involves double injection of a carefully calculated, reduced amount FeDex on day 3 and 14 after birth. Although this procedure has been shown to achieve the objectives, its application in the practice is questionable due to labor considerations at farms [59].

### 2.2. Oral Supplementation

“Supplementation” with soil iron*.* Rooting is a natural behavior for pigs frequently used at all ages. Pigs root in the soil in different ways for different reasons including searching for food. Iron is a relatively abundant element (20–40 g/kg) of the soil [60], which is considered a valuable source of this nutrient (mineral) for wild boar piglets living in their natural woodland habitat. In contrast, modern indoor farming systems prevent contact of piglets with soil and thus deprive them access to this source of iron. It has been suggested that in outdoor production iron supplementation of piglets occurs at least in part by ingesting this microelement from the soil [61]. However, experiments conducted on a large number of piglets (more than 2300 per group) clearly showed that outdoor-reared animals still need parenteral iron supplementation with FeDex, otherwise they develop anemia (Hgb level—5.1 g/dL) and show increased mortality [21]. Other studies demonstrated that environmental iron, ingested from the soil plays an essential role in maintaining correct iron status in domestic piglets during the pre-weaning period [62].

Iron supplements as feed additives. Over the years, various dietary iron supplements such as iron salts [63], iron chelates [13], carbonyl iron [64], iron polymaltose [65], and iron microparticles [66] have been used to prevent/treat IDA in piglets. Despite such huge diversity of oral iron supplements, there is a general consensus that dietary supplementation with iron is less efficient in rectifying hematological status of piglets compared with parenteral one (reviewed in [10]). On the other hand, supplementation *per os*, when it is based on voluntary intake of feed containing supplemental iron, it is a non-stressful, less time-consuming procedure, which avoids possible risks of iatrogenic disease transmission. However, low consumption of solid feed or drinking water by piglets during the first two weeks after birth strongly reduces the therapeutic efficiency of this strategy despite using various feeding devices and chemical attractants [17,67]. Although applying of excessive doses of supplemental iron added to the feed has been shown to improve iron status in piglets at a rate comparable to FeDex injection [67], the usefulness of this method seems to be doubtful because of strong adverse side effects observed mainly at the level of gastrointestinal tract and liver [68]. Regarding the oral route, iron can be also individually delivered to piglets in the form of a paste directly into the mouth [69]. In this case, the amount of administered iron can be tightly controlled. However, reports on the labor consumption of this procedure and its influence on the welfare of supplemented animals are controversial [70,71].

Despite several limitations in oral supplementation of piglets with iron, the search is ongoing for new iron supplements characterized by high bioavailability and potentially overcoming canonical pathways of iron absorption. In some sense, heme is a good candidate for such new although already long known iron supplement. Heme, a ferrous iron protoporphyrin IX complex, is employed as a prosthetic group in diverse proteins (including hemoglobin) that participate in important biological processes [72]. On one side, dietary heme uptake has been recognized for more than 60 years [73] and many studies have since confirmed that in mammals (except from mice [74]) absorption of heme is far more efficient than that of inorganic iron [73]. Accordingly, heme iron has been successfully used as iron supplement to treat iron deficiency in humans [75,76]. On the other hand, the use of heme iron to prevent IDA in piglets has not attracted much interest from pig breeders. Nevertheless, some studies [17,66,77] including our own [17] clearly showed that supplementation of piglets based on voluntary intake of hemoglobin from the feed can rescue these animals from severe IDA (piglets maintained hemoglobin level at 8 g/dL throughout the experimental period i.e., from day 3 to day 28 after birth) despite the reduced intake of solid feed during the first 10 days postpartum [17]. Importantly, dietary supplementation with hemoglobin promoted combating of severe IDA in piglets without inducing a disadvantageous increase in hepcidin expression [17]. We proposed that the well-known high bioavailability of heme iron may depend on a split pathway mediating the transport of heme-derived elemental iron as well as intact heme from the interior of duodenal enterocytes to the bloodstream.

The use of encapsulation technologies has been proposed not only to improve bioavailability of supplemental iron delivered by the oral route [78] but also to decrease its adverse effects in the gastrointestinal tract, due to the oxidative toxicity of ferrous iron [68]. Some of the novel oral iron supplements developed by encapsulating in various kinds of matrix inorganic and heme iron have been tested on anemic piglets and found to improve RBC indices [66,79]. Among encapsulation technologies, liposomes, bilayer phospholipid vesicles, have attracted much interest as efficient drug delivery systems [80]. In our recent studies we successfully used liposomal iron (Sideral^®^Pharmanutra, Pisa, Italy), a new generation iron (ferric phosphate) supplement containing phospholipids and sucrose esters of fatty acids, assuring its high bioavailability and tolerability [81]. The efficacy of this drug in correcting iron deficiency attested by the restoration of physiological hemoglobin levels has been proven in human and animal studies [82,83]. We show that liposomal iron given orally to suckling piglets is a suitable feed additive for the reinforcement of iron status in piglets in the critical period from birth to weaning. Oral supplementation with liposomal iron is not only as effective as the parenteral one with FeDex, but in addition it seems to be less toxic [55].

## 3. Concluding Remarks

**Figure 2:**
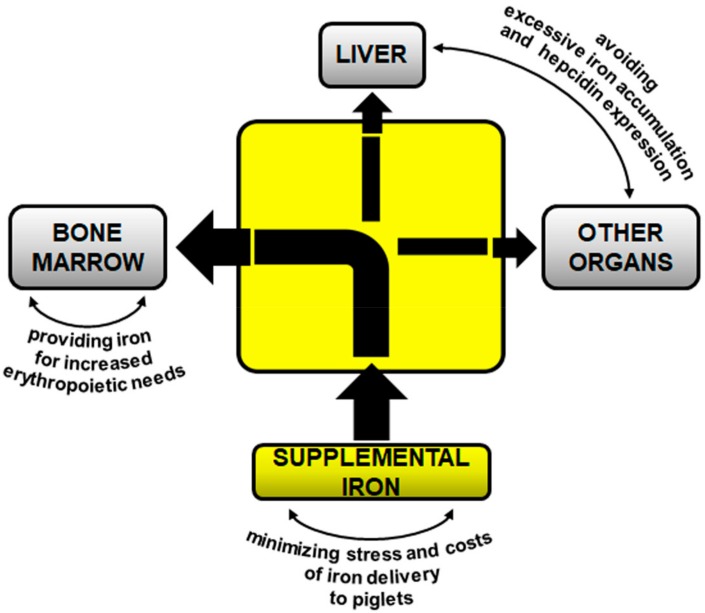
Main objectives of sustainable iron supplementation in suckling piglets. See description in the text for details.

Intramuscular administration of large amounts of FeDex to suckling piglets is long-established and commonly considered most favorable procedure used for preventing/treatment of IDA in the neonatal/early postnatal period. Here, we propose reconsidering this routine supplementation in the light of recent advances in molecular understanding of homeostatic regulation of iron in mammals [84], including mammalian neonates [85,86]. Our concept of optimal prophylaxis/treatment of IDA in suckling piglets is outlined in Figure 2. The main objective of iron supplementation is to meet current needs of this microelement for erythropoiesis, a physiological process consuming large amounts of iron. It seems that rebuilding of depleted hepatic iron stores of suckling piglets with supplemental iron is physiologically less important and plays a secondary role. The process of replenishment of iron reservoirs should proceed gradually and be driven by dietary iron contained in the basal feed routinely given to piglets during rearing. This approach will allow the reduction of the amount of supplemental iron administered to piglets. Lower dose iron given to piglets will primarily decrease its toxicity but will also minimize hepcidin expression and in consequence, maximize both natural iron absorption from the gut and supply of iron recovered from reticuloendothelial macrophages. Finally, when providing exogenous iron to piglets, we cannot leave aside neither the economical aspect of this procedure nor piglet welfare. In this context, oral supplementation based on voluntary intake of iron compounds added to the feed seems to be an optimal solution. However, major obstacles standing on this way are poor intake of solid feed by piglets during the first two weeks after birth, and neonatal immaturity of the piglets’ absorption molecular mechanisms. For this reason, there is a need to test the therapeutic efficacy of new iron supplements such as iron nanoparticles and encapsulated iron compounds overcoming canonical pathways of iron absorption. Importantly, considering that pig is a recognized model for human nutrition [87,88], we propose that piglet model of “physiological” IDA may be useful in preclinical studies for testing various iron food supplements in humans.
