# Correction: Mateusz, S., et al. Iron Supplementation in Suckling Piglets: An Ostensibly Easy Therapy of Neonatal Iron Deficiency Anemia. *Pharmaceuticals* 2018, *11*, 128

**DOI:** 10.3390/ph12010022

**Published:** 2019-01-29

**Authors:** Mateusz Szudzik, Rafał R. Starzyński, Aneta Jończy, Rafał Mazgaj, Małgorzata Lenartowicz, Paweł Lipiński

**Affiliations:** 1 Department of Molecular Biology, Institute of Genetics and Animal Breeding, Polish Academy of Sciences, Jastrzębiec, 05-552 Magdalenka, Poland; m.szudzik@ighz.pl (M.S.); r.starzynski@ighz.pl (R.R.S.); a.jonczy@ighz.pl (A.J.); r.mazgaj@ighz.pl (R.M.); 2 Department of Genetics and Evolution, Institute of Zoology, Jagiellonian University, Gronostajowa 9, 30-387 Kraków, Poland; malgorzata.lenartowicz@gmail.com; 3 Department of Genetics and Animal Breeding, Poznan University of Life Sciences, Wołyńska 33, 60-637 Poznań, Poland

The authors wish to make the following corrections to this paper [1]: the term “liposomal” should be replaced with the term “sucrosomial” in the following places:

1. On page 7, line 4 from the bottom;
2. On page 8, line 1 from the top;
3. On page 8, line 3 from the top.

The authors would like to apologize for any inconvenience caused to the readers by these changes.
